# Survey of methadone-drug interactions among patients of methadone maintenance treatment program in Taiwan

**DOI:** 10.1186/1747-597X-7-11

**Published:** 2012-03-20

**Authors:** Hsin-Ya Lee, Jih-Heng Li, Li-Tzy Wu, Jin-Song Wu, Cheng-Fang Yen, Hsin-Pei Tang

**Affiliations:** 1 School of Pharmacy, College of Pharmacy, Kaohsiung Medical University, No.100 Shih-Chuan 1st Road, Kaohsiung City 807, Taiwan; 2 Ph.D. Program in Toxicology, College of Pharmacy, Kaohsiung Medical University, No.100 Shih-Chuan 1st Road, Kaohsiung City 807, Taiwan; 3 Department of Psychiatry and Behavioral Sciences, Duke University Medical Center, Durham, NC 27710, USA; 4 Department of Health, Executive Yuan, Kao-ping Division, Bureau of National Health Insurance, Executive Yuan, No.157, Jiu-Ru 2nd Road, Kaohsiung City 807, Taiwan; 5 Department of Psychiatry, Kaohsiung Medical University Chung-Ho Memorial Hospital, No.100 Shih-Chuan 1st Road, Kaohsiung City 807, Taiwan; 6 Department of Psychiatry, Faculty of Medicine, College of Medicine, Kaohsiung Medical University, No.100 Shih-Chuan 1st Road, Kaohsiung City 807, Taiwan; 7 Department of Addiction and Forensic Psychiatry, Jianan Mental Hospital, No.80, Ln. 870, Jhong-Shan Road, Rende Dist., Tainan City 71742, Taiwan

**Keywords:** Methadone maintenance program, Methadone-drug interactions, Benzodiazepines, QTc prolongation effect, Adverse drug reactions or ADR

## Abstract

**Background:**

Although methadone has been used for the maintenance treatment of opioid dependence for decades, it was not introduced in China or Taiwan until 2000s. Methadone-drug interactions (MDIs) have been shown to cause many adverse effects. However, such effects have not been scrutinized in the ethnic Chinese community.

**Methods:**

The study was performed in two major hospitals in southern Taiwan. A total of 178 non-HIV patients aged ≥ 20 years who had participated in the Methadone Maintenance Treatment Program (MMTP) ≥ 1 month were recruited. An MDI is defined as concurrent use of drug(s) with methadone that may result in an increase or decrease of effectiveness and/or adverse effect of methadone. To determine the prevalence and clinical characteristics of MDIs, credible data sources, including the National Health Insurance (NHI) database, face-to-face interviews, medical records, and methadone computer databases, were linked for analysis. Socio-demographic and clinical factors associated with MDIs and co-medications were also examined.

**Results:**

128 (72%) MMTP patients took at least one medication. Clinically significant MDIs included withdrawal symptoms, which were found among MMTP patients co-administered with buprenorphine or tramadol; severe QTc prolongation effect, which might be associated with use of haloperidol or droperidol; and additive CNS and respiratory depression, which could result from use of methadone in combination with chlorpromazine or thioridazine. Past amphetamine use, co-infection with hepatitis C, and a longer retention in the MMTP were associated with increased odds of co-medication. Among patients with co-medication use, significant correlates of MDIs included the male gender and length of co-medication in the MMTP.

**Conclusions:**

The results demonstrate clinical evidence of significant MDIs among MMTP patients. Clinicians should check the past medical history of MMTP clients carefully before prescribing medicines. Because combinations of methadone with other psychotropic or opioid medications can affect treatment outcomes or precipitate withdrawal symptoms, clinicians should be cautious when prescribing these medications to MMTP patients and monitor the therapeutic effects and adverse drug reactions. Although it is difficult to interconnect medical data from different sources for the sake of privacy protection, the incumbent agency should develop pharmacovigilant measures to prevent the MDIs from occurring. Physicians are also advised to check more carefully on the medication history of their MMTP patients.

## Background

Methadone, a long-acting synthetic opioid originally developed for pain control, is now mainly used for the treatment of opioid dependence [1]. The Methadone Maintenance Treatment Program (MMTP), by providing adequate oral methadone doses to heroin-dependent patients once a day, aims to reduce cravings for heroin, injecting drug use behavior, risks of HIV or hepatitis infection, criminal activity, and eventually to improve their quality of life [2-6]. However, several factors, including methadone dosage [7-10], adverse drug reactions (ADRs), and methadone-drug interactions (MDIs) [11,12], can affect treatment compliance. Receipt of low or inadequate doses of methadone has been associated with higher rates of withdrawal symptoms and dropout rates [13,14]. In contrast, overmedication may cause somnolence, itching, hypotension, or even respiratory depression [15]. Moreover, methadone-associated ADRs, including constipation, nausea, erectile dysfunction [16], sleeping disorders, menstrual cycle irregularities [17], and in severe cases, disturbance of patients' daily lives, are often observed. The most dangerous side effect of methadone is torsade de pointes (TdP) [18-22], which may threaten a patient's life. MDIs can either increase [23] or decrease [24] serum methadone levels, leading to treatment failure or even death [19,25]. Therefore, proper methadone dose adjustment and therapeutic drug monitoring should be handled with caution when MMTP patients also have received other medicines that could yield TdP or interfere with the activities of cytochrome P 450 (CYP450) enzymes, such as 3A4 [26], 2B6 [26,27], 2C19 [27] or 2D6 [28].

Previous studies have revealed that mortality of MMTP patients due to methadone overdose is frequently associated with drug combinations, such as benzodiazepines (59%), opiates (86%), or cocaine (34%) [29]. Case reports have also described methadone-associated death due to co-administration of methadone with temazepam and amitriptyline [18], or with sodium valproate, amitriptyline, and fluoxetine [19]. In addition, methadone-antiretroviral agent interactions have been investigated. For instance, nevirapine can significantly reduce the methadone plasma concentration [30]. QT prolongation has been observed in patients who take methadone with abacavir, nevirapine, tenofovir, and voriconazole [31]. However, most patients participated in these studies were from Western societies and, therefore, are limited to Caucasian or African origins [32-35]. In addition, many, if not most, studies focused on the interactions of anti-retroviral agents and methadone among HIV patients in MMTP [24,29-31,34-36]. Furthermore, these studies were mostly based on reviews of medical or pharmacy records, and therefore may be limited by incomplete information and recall bias. Because MMTP was not implemented in China or Taiwan until 2004 and 2006, respectively, few, if any, studies have been conducted to assess the prevalence of MDIs in non-HIV MMTP patients of Chinese origin.

In Taiwan, the prevalence of IDUs among all addiction treatment admissions increased from 34.7% in 2000 to 63.9% in 2004, and the percentage of needle-sharing among IDUs increased from 4.0% in 2000 to 15% in 2004 [37]. To address the escalating IDU and HIV infections through needle-sharing, the first MMTPs were implemented in July 2006 [38,39]. Because of this new measure, it is especially important for medical professionals to understand the comprehensive effects of MDIs among MMTP patients to inform treatment planning and care management.

The National Health Insurance (NHI) program, a national universal health insurance program with a coverage rate of over 99% for all citizens in Taiwan, provides general medical and pharmacy records for all patients. However, the MMTP in Taiwan is a separate treatment entity from general medical practice. Unlike the general medical care that is covered by the national health insurance and eligible to all citizens, the MMTP is subsidized from the budget of Taiwan Centers for Disease Control (TCDC) and applied only to patients enrolled in the program. The teamwork of MMTP includes psychiatrists with certified addiction treatment specialty, nurses and case managers who assist the patients in finding the social cares for clients or monitoring the days of attendance at MMTP, and pharmacists who dispense methadone and check whether patients have taken the medicines. The MMTP also provides educational programs for specific patients who were under the conditions of deferred prosecution.

In order to prevent MMTP patients from the potential danger of clinically significant MDIs, it is important to identify the prevalence and relevance of MDIs with clinically adverse effects. The clinical evidence will assist TCDC in revising guidelines to improve the drug safety among MMTP clients. Therefore, we utilized multiple sources of available data (NHI database, face-to-face interviews, medical records, and methadone computer databases) to investigate the prevalence and clinical relevance of MDIs in non-HIV patients.

## Methods

### Data sources and study sample

This study was conducted from February 2010 to October 2010 at Jianan Mental Hospital of the Department of Health, the first mental hospital to implement a MMTP, and Chung-Ho Memorial Hospital of Kaohsiung Medical University, a major medical center in southern Taiwan. One-hundred seventy-eight patients with non-HIV infections (confirmed by medical records) aged ≥ 20 years who had participated in the MMTP ≥ 1 month were recruited. An MDI was defined as concurrent use of drug(s) with methadone that may result in an increase or decrease of effectiveness and/or adverse effect of methadone. Medical expenses of HIV-positive patients in Taiwan are covered by the national health insurance program and exempt from medical co-payments to encourage their use of treatments. They hold a medical registration card different from non-HIV patients for medical exemption when seeking treatments. For reasons of confidentiality and possible sampling bias, HIV-positive patients were excluded in this study.

All information was kept strictly confidential and used for research proposes only. Participants received an indemnity of about 8.5 US dollars for participating in this study. The study was approved by the Institutional Review Boards of Jianan Mental Hospital (Approval No. 10-002) and Chung-Ho Memorial Hospital (Approval No. KMUH-IRB-980429), and written informed consent was obtained from all participants.

Socio-demographic characteristics and histories of substance use were obtained from structured face-to-face interviews, which were carried out in a private space within the clinic away from other staff and patients by a trained research assistant using a structured questionnaire. Socio-demographic characteristics included age, sex, educational level (years of education completed), current marital status, current employment status, and sources of financial support. Substance use referred to the use of cigarettes, alcohol, or betel quid, while drug use included illicit use of heroin, amphetamines (including methamphetamine), ketamine, MDMA (3,4-methylenedioxymethamphetamine; ecstasy), or benzodiazepines. The definition for use of these substances was based on epidemiological evidence from national surveys of substance use in Taiwan [40]. Data regarding daily attendance records and methadone doses were obtained from the computer database of the TCDC - a centralized data depository for the national MMTP program. Baseline demographic characteristics, admission date to the MMTP, and data of hepatitis, including hepatitis B (HBV) or hepatitis C (HCV), as defined by a patient's serological blood test with the presence of hepatitis B surface antigen (HBsAg) or detected antibodies to HCV, respectively, were derived from medical records.

### Prescription data

In March 1995, the NHI was initiated as a national universal health insurance program for all citizens in Taiwan; by December 2010, up to 99.5% of the citizens in Taiwan had participated in the program. All inpatient and outpatient prescription data from all registered participants in the NHI data depository from the time that MMTP was initiated in July 2007 to August 2010 were available for research.

### Methadone-drug interactions (MDIs) data

The severity levels of MDIs, based on Micromedex^® ^[41], Lexi-Interact™ [42] and published studies [43,44], are summarized in Table 1. In Micromedex^®^, it is categorized into five levels by the severity, namely, contraindicated, major, moderate, minor and unknown, while the severity of DDIs in Lexi-Interact™ is categorized into three levels, i.e., major, moderate, and minor. Because some MDIs were not listed in these two databases, we also incorporated the severity of MDIs from published reports [43,44].

**Table 1:** Severity levels of potential methadone-drug interactions (MDIs) based on Micromedex^® ^and Lexi-Interact™ interaction databases

Level	Definition	Drugs
***1***^***a***^	Drugs should not be coadministered as they might lead to serious adverse events or precipitate opioid withdrawal	Buprenorphine, Tramadol, Nalbuphine, Naloxone, Naltrexone, Amiodarone, Butorphanol, Ciprofloxacin, Chlorpromazine, Cisapride, Dezocine, Dofetilide, Droperidol, Dronedarone, Fentanyl/Droperidol, Fluphenazine, Fospropofol, Gatifloxacin, Halofantrine, Haloperidol, Ibutilide, Iloperidone, Lapatinib, Levofloxacin, Mesoridazine, Moxifloxacin, Nalbuphine, Nilotinib, Paliperidone, Perphenazine, Pimozide, Prochlorperazine, Promazine, Promethazine, Quinidine, Quinine, Ranolazine, Sotalol, Sunitinb, Tapentadol, Telithromycin, Tetrabenazine, Thiethylperazine, Thioridazine, Trifluoperazine, Vardenafil
***2***^***b***^	A potential interaction might modify the dosage; monitor closely to minimize clinical consequences	Alprazolam, Estazolam, Flurazepam, Midazolam, Zopiclone, Clormethiazole, Methylphenidate, Amitriptyline, Desipramine, Imipramine, Nortriptyline, Protriptyline, Phenobarbital, Dexamethasone, Fusidic acid, Rifampicin, Spironolactone, Diltiazem, Cimetidine, Dihydroergotamine, Fluconazole, Ketoconazole, Erythromycin, Clarithromycin, Moclobemide, Fluoxetine, Fluvoxamine, Paroxetine, Sertraline, Risperidone, Merperidine, Alfentanil, Propoxyphene, Morphine, Carbamazepine, Phenytoin
***3***^***c***^	Minor or unknown interactions	Dextromethorphan, Nifedipine, Diazepam, Metronidazole, Omeprazole, Verapamil

^a^The severity of the MDI was evaluated as a "contraindication" or "major" in Micromedex^® ^and Lexi-Interact™. ^b^A potential MDI may lower or raise serum methadone levels, or drugs that may result in altered metabolism or unpredictable interactions in combination with methadone. ^c^A potential DDI may result in minor or unknown interactions

Therefore, the criteria for the severity of an interaction is integrated and defined as: *level 1*: drugs should not be co-administered or major MDIs should be considered, as stated in Micromedex^® ^[41] and Lexi-Interact™ [42], because they may lead to serious ADRs or precipitate opioid withdrawal; *level 2*: drugs may have potential interactions that can modify the dosage, and patients should be monitored closely to minimize adverse clinical consequences; and *level 3*: minor or unknown interactions. The patients' clinical information, including data collected from medical records, NHI and CDC-MMTP, was connected by personal ID with a written informed consent of the patient. These three databases were incorporated by Microsoft Access 2003. To link the data with the Anatomical Therapeutic Chemical (ATC) codes in the NHI dataset, drugs listed in Table 1 were recorded on the basis of the ATC classification system. This connection was used to screen each patient's medication profile from the time of MMTP initiation to August 2010. When an MDI was identified, the interacting drug, the severity level, and the pharmacological class were recorded.

### Statistical analyses

Descriptive statistics were performed to examine the interacting drugs and severity levels. Baseline socio-demographic characteristics, duration of membership in the MMTP, hepatitis data, substance use status, as well as data on daily methadone attendance records and methadone doses, were compared using the Mann-Whitney *U *test for continuous variables and the Chi-square test or Fisher's exact test for categorical variables to account for the small sample size. Logistic regression was conducted to explore associations of potentially predictive variables (socio-demographics, duration of membership in the MMTP, clinical data, and substance use characteristics) with co-medication and MDIs.

All analyses were performed using the JMP software version 9.0 (SAS Institute, Cary, NC, USA); *p*-values were two-sided with statistical significance set at *p *< 0.05.

## Results

### Selected characteristics of MMTP patients

The 178 participants included 156 males and 22 females, with a mean age of 39.5 ± 7.1 years (range: 25-59 years) and a mean weight of 68.8 ± 12.8 kg (40-118 kg). The mean methadone daily maintenance dose was 50.8 ± 30.5 mg (5-250 mg/d). The prevalence of HBV and HCV was 18.5% and 89.9%, respectively. The drug interaction group had a higher retention in the MMTP than those not in the drug interaction group (22.8 vs. 17.7 months, P = 0.04, df = 126; Table 2); there were no significant differences in the other characteristics by drug interaction status (presence or absence of MDIs).

**Table 2:** Selected characteristics of co-medication subpopulation in the MMTP (128 patients) according to the presence or absence of methadone-drug interactions (MDIs)

	No interaction (n = 43)	Drug interaction (n = 85)	*df*	*P*-value*
Mean age, (SD)	40.7 (6.8)	39.0 (7.4)	127	0.20
Male, *n *(%)	36 (83.7)	75 (88.2)	1	0.47
Mean body mass index, kg/m^2 ^(SD)	23.6 (3.3)	24.4 (3.9)	125	0.24
Education, *n *(%)				
Less than high school	26 (60.5)	47 (55.3)	1	0.58
High school or above	17 (39.5)	38 (44.7)		
Marital status, *n *(%)				
Married or living with partner	13 (30.2)	27 (32.1)	2	0.69
Never married	24 (55.8)	41 (48.8)		
Divorced/widowed	6 (14.0)	16 (19.1)		
Employment, *n *(%)				
Employed	30 (69.8)	55 (64.7)	1	0.57
Other	13 (30.2)	30 (35.3)		
Current illicit drug use, *n *(%)				
Heroin	22 (51.2)	30 (35.3)	1	0.08
Amphetamine(s)	1 (2.3)	3 (3.5)	1	0.99
Benzodiazepine(s)	7 (16.3)	14 (16.5)	1	0.98
Heroin use years, (SD)	9.5 (6.3)	7.6 (5.1)	119	0.11
Drug use in the past, *n *(%)				
Heroin	43 (100)	85 (100)	1	--
Amphetamine(s)	26 (60.5)	56 (65.9)	1	0.55
MDMA	0 (0.0)	8 (9.4)	1	0.05
Ketamine	1 (2.3)	10 (11.8)	1	0.10
Other substance use, *n *(%)				
Cigarettes	33 (83.7)	73 (85.9)	1	0.75
Alcohol	13 (30.2)	30 (35.3)	1	0.57
Betel quid	9 (20.9)	28 (32.9)	1	0.16
HCV coinfection, *n *(%)	42 (97.7)	79 (92.9)	1	0.42
HBV coinfection (HBsAg-positive), *n *(%)	9 (20.9)	18 (21.2)	1	0.97
Current methadone dose, mg (SD)	53.9 (25.7)	53.0 (29.7)	127	0.86
MMTP participation period, months (SD)	17.7 (12.5)	22.8 (13.8)	126	0.04*

*: statistical significance set at *p *< 0.05; Comparisons performed by the Mann-Whitney *U *test, Chi-square test or Fisher's exact test when appropriate

### Methadone-drug interactions

**Figure 1:**
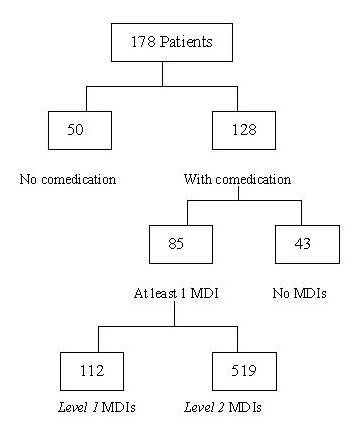
**Prevalence of identified methadone-drug interactions (MDIs)**. Of the 178 participants, 48% (85/178) had at least one MDI event. A total of 112 MDI events were classified as *level 1*, which indicated that drugs should not be coadministered with methadone, as they may lead to serious adverse reactions or precipitate opioid withdrawal.

**Figure 2:**
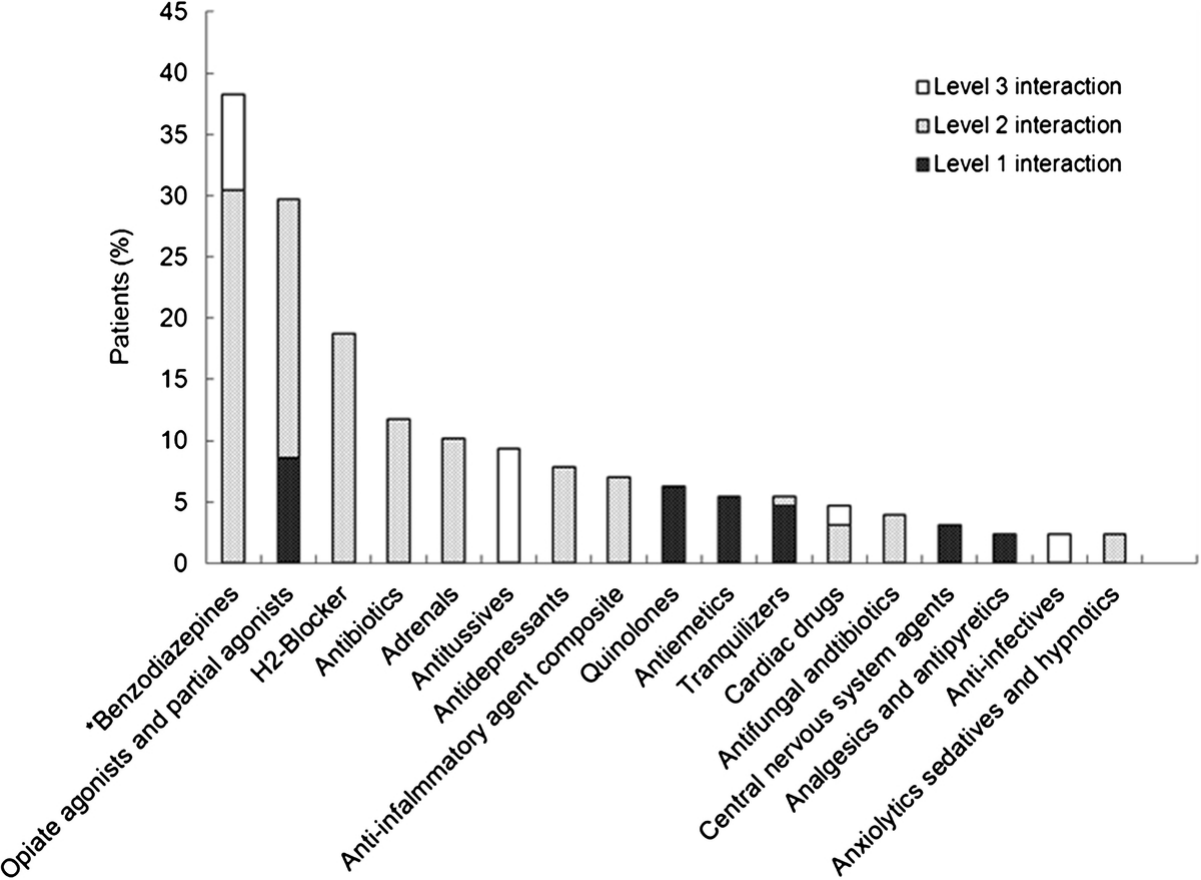
**Occurrences of MDI among 85 out of the 178 MMTP patients**. The bars show the percentages of patients taking different classes of drugs and their corresponding pharmacological levels. *In Taiwan's National Health Insurance (NHI) program, the pharmacologic classification is based on the Anatomical Therapeutic Chemical (ATC) system in general. According to the ATC system, benzodiazepines are not included in tranquilizers. Rather, they are included in anxiolytics. However, in the NHI program, benzodiazepines are counted separately from other anxiolytics. Therefore, benzodiazepines, anxiolytics and tranquilizers are grouped separately.

Overall, there were 7,239 co-medication data that 72% (n = 128) of MMTP patients were on at least one medication. Comparatively common medications co-existing with methadone were acetaminophen (7%), flunitrazepam (5%), diclofenac (2.5%), zolpidem (2.4%), and trazodone (2.3%). Twenty-six MMTP patients received pain medications, such as morphine, tramadol, buprenorphine, and dextropropoxyphene. Thirty-six patients had co-occurring anxiety/depressive disorders and were dependent on benzodiazepine and benzo-like medications. Ten patients had depressive disorders and took other antidepressants, such as paroxetine, amitriptyline, imipramine, fluoxetine and sertraline. There were a total of 699 MDI events, and 85 (48%) participants had at least 1 MDI (Figure 1). Frequent therapeutic classes of MDIs are shown in Figure 2. The three most common MDIs pharmacological classes were benzodiazepines (38.1%), opiate agonists and partial agonists (29.7%), and H_2_-Blocker (18.8%). MDI pharmacological classes belonging to the most dangerous level (*level 1*) included opiate agonists and partial agonists (8.6%), quinolones (6.3%), antiemetics (5.5%), tranquilizers (4.7%), central nervous system (CNS) agents (3.1%), and analgesics and antipyretics (2.3%).

The five comparatively common MDIs (Table 3) included alprazolam, cimetidine, dexamethasone, tramadol, and estazolam. Interactions of benzodiazepines with methadone, including alprazolam, estazolam, midazolam (2.7%), and diazepam (2.1%), were also observed in some patients.

**Table 3:** Twenty most frequently observed methadone-drug interactions (MDIs) among co-medication subpopulation in the MMTP (128 patients)

Drugs that interact with methadone	Level	n (%)	Mechanisms of MDI
Tramadol	1	42 (6.0)	Concomitant administration of methadone and tramadol may result in withdrawal symptoms; methadone (moderate CYP 2D6 inhibitor) may decrease the metabolism of tramadol
Chlorpromazine	1	22 (3.1)	The concomitant use of methadone and chlorpromazine may cause additive CNS and respiratory depression
Levofloxacin	1	16 (2.3)	Levofloxacin may increase the QTc prolonging effects of methadone
Prochlorperazine	1	12 (1.7)	The concomitant use of methadone and prochlorperazine may cause additive CNS and respiratory depression
Alprazolam	2	99 (14.2)	Alprazolam may cause additive CNS depression
Cimetidine	2	84 (12.0)	Cimetidine (moderate CYP 3A4 and 2D6 inhibitor) may decrease the metabolism of methadone, raise serum methadone concentrations and consequently increase the toxicity of methadone
Dexamethasone	2	48 (6.9)	Dexamethasone (moderate CYP 3A4 and 2B6 inducer) may increase the metabolism of methadone, lower serum methadone concentrations and result in withdrawal symptoms
Estazolam	2	40 (5.7)	Estazolam may cause additive CNS depression
Fusidic acid	2	26 (3.7)	Fusidic acid may induce CYP enzyme
Pethidine	2	25 (3.6)	Interaction probably occurs due to additive opioid effects
Diltiazem	2	23 (3.3)	Diltiazem (moderate CYP 3A4 inhibitor) may decrease the metabolism of methadone, raise serum methadone concentrations and consequently increase the toxicity of methadone
Carbamazepine	2	22 (3.1)	Carbamazepine (strong CYP 2B6 inducer) may increase the metabolism of methadone, lower serum methadone concentrations and result in withdrawal symptoms
Imipramine	2	22 (3.1)	Imipramine (moderate CYP 2D6 inhibitor) may decrease the metabolism of methadone; combination with methadone increases tricyclic antidepressant (TCA) toxicity
Risperidone	2	20 (2.9)	Risperidone accelerates methadone metabolism via interfering with absorption or displacing methadone from plasma protein binding sites and results in withdrawal symptoms
Midazolam	2	19 (2.7)	Midazolam may cause additive CNS depression
Nifedipine	3	18 (2.6)	Methadone possibly increases the effects of nifidepine and increase the toxicity of nifedipine
Morphine	2	13 (1.9)	Interaction probably occurs due to additive opioid effects
Paroxetine	2	12 (1.7)	Paroxetine (moderate CYP 2B6 and 2D6 inhibitor) may decrease the metabolism of methadone and raise serum methadone concentrations and consequently increase the toxicity of methadone
Erythromycin	2	12 (1.7)	Erythromycin (CYP 3A4 inhibitor) may decrease the metabolism of methadone, raise serum methadone concentrations and consequently increase the toxicity of methadone
Dextromethorphan	3	25 (3.6)	Methadone may increase the levels/effects of dextromethorphan and increase the toxicity of dextromethorphan
Diazepam	3	15 (2.1)	Diazepam may increase the methadone effects and consequently increase the toxicity of methadone

Level 1 MDIs may lead to serious ADRs or precipitate opioid withdrawal [41,42]. The drugs resulting in *level 1 *MDIs with the most clinical significance are shown in Table 4.

**Table 4:** Drugs with Level-1 MDIs with the most clinical significance

Drugs	Frequency	Mechanisms of MDI
Tramadol	42	Concomitant administration of methadone and tramadol may result in withdrawal symptoms; methadone (moderate CYP 2D6 inhibitor) may decrease the metabolism of tramadol
Chlorpromazine	22	The concomitant use of methadone and chlorpromazine may cause additive CNS and respiratory depression
Levofloxacin	16	Levofloxacin may increase the QTc prolonging effects of methadone
Prochlorperazine	12	The concomitant use of methadone and prochlorperazine may cause additive CNS and respiratory depression
Moxifloxacin	6	Moxifloxacin may increase the QTc prolonging effects of methadone
Ciprofloxacin	5	Ciprofloxacin may increase the QTc prolonging effects of methadone
Haloperidol	5	Haloperidol may increase the QTc prolonging effects of methadone
Buprenorphine	1	Concomitant administration of methadone and buprenorphine may result in withdrawal symptoms
Droperidol	1	Droperidol may increase the QTc prolonging effects of methadone
Nalbuphine	1	Concomitant administration of methadone and nalbuphine may result in withdrawal symptoms
Thioridazine	1	The concomitant use of methadone and thioridazine may cause additive CNS and respiratory depression

Of the identified MDIs, one significant and dangerous side effect of methadone was a severe QTc prolongation effect, which may be caused by haloperidol, ciprofloxacin, droperidol, levofloxacin, or moxifloxacin. The MDIs that could produce withdrawal symptoms were found in MMTP patients co-administered with buprenorphine, nalbuphine, or tramadol. Another hazardous effect was additive CNS and respiratory depression, which could result from methadone in combination with chlorpromazine, prochlorperazine, and thioridazine.

### Logistic regression of correlates of coadministration

Adjusted logistic regression analysis showed that past amphetamine use (Adjusted Odds Ratio, AOR = 2.68, *P *= 0.03), HCV infection (AOR = 6.68, *P *= 0.01), HBV infection (AOR = 3.08, *P *= 0.05) and a longer duration in the MMTP (AOR = 1.08, *P *< 0.0001) were associated with increased odds of co-medication (Table 5) and that being single or divorced/widowed were associated with reduced odds of co-medication. Compared with male patients (AOR = 0.63, *P *= 0.54), female patients were associated with a higher risk of having a co-medication.

**Table 5:** Factors associated with comedication among the total population in the MMTP (178 patients)

Characteristic	Adjusted OR (95% CI)	*P-value*
Age	1.02 (0.95 ~ 1.09)	0.61
Sex		
Female	1.00	-
Male	0.63 (0.13 ~ 2.61)	0.54
Education		
Less than high school	1.00	-
High school or above	2.42 (1.00 ~ 6.25)	0.06
Marital status		
Married or living with partner	1.00	-
Never married	0.23 (0.06 ~ 0.72)	0.02*
Divorced/widowed	0.24 (0.06 ~ 0.89)	0.03*
Employment		
Employed	1.00	-
Other	1.06 (0.41 ~ 2.66)	0.91
Drug use in the past		
Amphetamine	2.68 (1.15 ~ 6.53)	0.03*
MDMA	0.19 (0.01 ~ 2.85)	0.26
Ketamine	4.84 (0.42 ~ 132.23)	0.25
Other use substances		
Cigarettes	1.43 (0.46 ~ 4.32)	0.53
Alcohol	1.01 (0.42 ~ 2.45)	0.98
Betel quid	1.44 (0.50 ~ 4.36)	0.50
HBV coinfection	3.08 (1.03 ~ 10.77)	0.05
HCV coinfection	6.68 (1.56 ~ 31.06)	0.01*
MMTP participation period	1.08 (1.04 ~ 1.12)	< 0.0001**

Logistic regression with all the other predictors entered into the model

**OR **= odds ratio

*: < 0.05; **: < 0.0001

### Logistic regression of correlates of MDIs

Stepwise logistic regression analysis was performed to identify associations of predictive variables with MDIs. Among patients with co-medication, adjusted analyses showed that significant correlates for MDIs were male (AOR = 4.88, *P *= 0.02), divorced/widowed (AOR = 2.24, *P *= 0.19), and a longer length of co-medication in the MMTP (AOR = 1.41, *P *= 0.002). However, current heroin use (AOR = 0.38, *P *= 0.04) was associated with reduced odds of MDIs (Table 6).

**Table 6:** Factors associated with methadone-drug interactions (MDIs) among the co-medication subpopulation in the MMTP (128 patients)

Characteristics	Adjusted OR (95% CI)	*P-value*
Age	0.94 (0.88 ~ 0.99)	0.07
Sex		
Female	1.00	-
Male	4.88 (1.25 ~ 20.17)	0.02*
Marital status		
Married or living with partner	1.00	-
Divorced/widowed	2.24 (0.71 ~ 7.94)	0.19
Current illicit drug use		
Heroin (yes vs. no)	0.38 (0.15 ~ 0.94)	0.04*
Number of comedications per MMTP participation months^a^	1.41 (1.17 ~ 1.79)	0.002*

Stepwise logistic regression analysis was performed to identify associations of predictive variables

^a^The number of comedications per MMTP participation months: MMT participation period for each patient divided by the total number of comedications

**OR **= odds ratio.

*: < 0.05; **: < 0.0001

Finally, a total of 6 ADR cases considered to have potential MDIs of *level 1 *or *2 *were identified from the prescription database (Table 7). Two cases exhibited symptoms of ADRs, such as depression and anxiety, while combining methadone with tramadol. Another two patients had received the 1^st ^generation antipsychotics (e.g., chlorpromazine, thioridazine) for psychotic disorders, and methadone-related ADRs were observed. The last two cases appeared to have ADRs associated with co-administration of methadone with dexamethasone, paroxetine, and ketoconazole, which are a CYP3A4 strong inducer, a CYP2B6 moderate inhibitor, and a CYP3A4 strong inhibitor, respectively.

**Table 7:** Suspected presence of Adverse Reactions (ADRs) in case reports resulting from methadone-drug interactions (MDIs)

**Case no**.	Suspected Drugs	Mechanisms	Description
1	Thioridazine	Additive CNS and respiratory depression	A 38-year-old man was diagnosed with drug-induced psychotic disorder with hallucinations and started on thioridazine, flunitrazepam, and trihexyphenidyl for three months. He then experienced anxiety causing hyperventilation, and was consequently treated with midazolam and oxazolam.
2	Chlorpromazine	Additive CNS and respiratory depression	A 31-year-old woman was started on chlorpromazine for insomnia. After three months, she was diagnosed with hyperventilation and tachycardia, which may have been a result of methadone or a methadone-chlorpromazine interaction.
3	Tramadol	May result in withdrawal symptoms	A 48-year-old man was started on tramadol for a month. He then felt anxious, which may have been caused by a methadone-tramadol interaction. Upon discontinuing tramadol, no other symptoms related to anxiety persisted.
	Dexamethasone	CYP3A4 strong inducer	This man was started on dexamethasone for intracranial injury. After coadministering methadone with dexamethasone for a month, he started to feel anxious.
4	Tramadol	May result in withdrawal symptoms	A 48-year-old man was prescribed tramadol for a month for fractures of the tibia and fibula. Then, he developed a depressive mood, which may be owing to a methadone-tramadol interaction.
5	Paroxetine	CYP2B6 moderate inhibitor	A 38-year-old man was diagnosed with depression and started on paroxetine for several months. When methadone was coadministered with paroxetine, an anxious feeling persisted.
6	Ketoconazole	CYP3A4 strong inhibitor	A 41-year-old man's foot was infected with mycoses and was started on ketoconazole for two months. He was then diagnosed with angina pectoris, which may be due to a methadone-ketoconazole interaction. Upon stopping ketoconazole, he did not experience any symptoms related to angina.

## Discussion

This was the first study that utilized multiple data sources to systematically examine methadone-drug interactions since the MMTP was implemented in Taiwan and to determine demographic and clinical correlates of comedication and MDIs among MMTP patients. Owing to the high coverage rate of the NHI (99%) in Taiwan, we were able to use the comprehensively collected national clinical data to characterize MDIs. Because the probability of MDIs and associated morbidities increases with polydrug use and some MDIs (e.g., an enhanced risk of overdose and poorer retention) may intensify poor outcomes among MMTP patients, it is clinically important to understand factors affecting MDIs in order to inform early detection and prevention of MDIs and to reduce adverse effects.

The majority of identified MDIs were associated with benzodiazepines (38.1%), the most-prescribed therapeutic drugs found in our participants. By comparison, higher proportions of patients (51.5% and 73.0%) were prescribed benzodiazepines in Switzerland [45] and Germany [46], respectively. Previous studies have shown that anxiety disorders were highly prevalent among opioid-dependent MMTP patients [47-49], and therefore many patients might have used benzodiazepines. Furthermore, MMTP patients who use or abuse benzodiazepines can have a negative clinical effect because they may have higher levels of addiction, use more illicit substances, be affected by other mental and social problems, and have difficulty in retaining in treatment [49,50]. Co-administration of benzodiazepines and methadone may increase the risk of methadone overdose or even death [18,51,52]. In this study, the most commonly prescribed benzodiazepine was alprazolam (14.2%), which is also frequently reported to be involved in methadone-related deaths [53,54].

Co-usage of opiate agonists and partial agonists (29.7%) with methadone was the second common pattern of use in this study. Combinations of methadone with buprenorphine, pethidine, and tramadol were also found in a study conducted in China [55], which are consistent with the results of this study. In particular, we found that morphine and pethidine were the most frequently prescribed opiate agonists among participants. The reason for co-administration may be a result of MMTP patients' surgical use or seeking of additional opiate analgesics to alleviate their craving. Patients in an opioid maintenance treatment program can receive buprenorphine or methadone, but normally they would not be prescribed simultaneously. However, a MMTP patient was prescribed with buprenorphine to relieve pain after surgery (Table 4). In addition, Manchikanti *et al.*[56] reported that a significantly increasing proportion of patients receiving controlled substances were revealed to use illicit drugs or other prescription opioids for nonmedical use. Therefore, clinicians should be cautious when MMTP patients have received opiate agonists, as they may enhance the risk of addictive effects or opioid toxicity [57].

Clinically significant MDIs may share one of the three common drug interaction mechanisms, which include (i) increasing the QTc prolonging effect, the most serious ADR of MDIs, which may consequently develop into fatal TdP [19-21]; (ii) enhancing addictive CNS and respiratory depression, which cause methadone-related deaths [19]; (iii) triggering opioid withdrawal symptoms, which may cause an increase in catecholamines plasma concentrations, leading to the development of stress cardiomyopathy [58] that, in turn, can cause death. Potential ADRs were also observed among patients who had MDIs in this study. In addition to the three mechanisms stated above, MDIs [27] caused by co-administering CYP3A4-inducing or CYP3A4-inhibiting agents should be carefully monitored and addressed. Moreover, studies [27,59,60] have suggested that CYP2B6 plays an important role in mediating methadone metabolism. Thus, caution should be taken when combining methadone with CYP2B6-inducing agents, such as carbamazepine, phenytoin, rifampin, and phenobarbital, or with CYP2B6-inhibiting agents, such as paroxetine, sertraline, and desipramine. However, because of the difference in the genetic polymorphism of *CYP*450 between Oriental and Caucasian populations, the results may not be generalized to those of the US and Western Europe.

The identified correlates for co-medication included use of amphetamines (e.g., methamphetamine) and co-infection with HCV. Poly-pharmacy or poly- substance use has been found to be common among MMTP patients, especially use of amphetamines and benzodiazepines [61]. On the other hand, chronic HCV infection may exhibit some symptoms, such as fatigue, nausea, loss of appetite, muscle ache, flu-like symptoms, and depression, or may even result in the development of cirrhosis or liver cancer [62], which may increase use of other illicit or non-prescribed drugs to relieve symptoms (e.g., self-medication). For instance, we found that 89.9% (160/178) of patients were infected with HCV, but only 3.4% (6/178) patients received HCV therapy (ribavirin plus peginterferon alpha 2A or 2B). A very low proportion of HCV treatment has been observed in MMTP patients [63].

The prevalence of cigarette use among MMTP patients was threefold the rate than that in the general population, and cigarette use increases the risk of morbidity and mortality [64]. Smoking is also associated with current mood or anxiety disorders among MMTP patients, especially in women [65]. In line with the literature, some sedatives or antidepressants were found to be frequently used in our MMTP patients, such as sertraline, paroxetine [23], fluoxetine, amitriptyline [66], and zolpidem [66], which may affect the serum methadone concentration due to the involvement of the same CYP450 enzymes with methadone metabolism. Further, methadone interacts not only with the above drugs, but also with nicotine, which may increase euphoria and decrease restlessness, irritability, and depression [67].

Identification of clinical factors associated with MDIs will assist physicians in eliminating or preventing potential risks. Male patients were strongly associated with having MDIs (AOR = 4.88, *P *= 0.02). Moreover, the odds of MDIs in patients with co-medications per MMTP participation months were 1.41 times greater than those without co-medications. Younger age was weakly associated with decreased odds of MDIs by 0.94. This can be explained in part by their lower rate of cardiovascular diseases, which are associated with serious ADRs (i.e., TdP) and are more prevalent among older patients [68]. In this study, some common ADRs, such as sweating, constipation, and insomnia, were noticed during the treatment, though two patients had been diagnosed with arrhythmia before this study, and one patient had tarchycardia related to a suspected methadone- chlorpromazine interaction in this study. Therefore, the prescription of medications that could induce QT interval prolongation should be avoided in these patients.

These results should be interpreted within the context of the following limitations. First, this study was conducted in southern Taiwan, and the results may not be generalized to other regions in Taiwan. Because MMTP patients may have different attitudes towards or habits related to the treatment of diseases, some may use over-the-counter (OTC) medications or Chinese herbal medicines to alleviate symptoms. Second, because OTC medications or Chinese herbal medicines are not covered by the national health insurance program, the interaction events resulting from OTC drugs-methadone or Chinese herbal medicines-methadone may be underestimated among MMTP patients who used additional OTC medications or Chinese herbal medicines. Moreover, flunitrazepam, a benzodiazepines drug, was not included in Micromedex^®^[41] or Lexi-Interact™[42]. Hence, we have not included this potential MDI in our list, and such MDIs may be underestimated. However, it should be noted that this kind of MDI may often be missed by clinicians. Finally, our definitions for

MDIs depend on the level of completeness of the available data banks (Micromedex^® ^and Lexi-Interact™). To minimize bias, several well-documented databases were also integrated in this study.

In conclusion, to improve clinical care and prevent MDIs and MDI-related deaths, it is important that frequent MDIs and their characteristics among MMTP patients are identified and incorporated into care management. The present study utilized multiple data sources (a national medical database, face-to-face interviews, medical records, and methadone computer databases) to systematically characterize MDIs to help inform clinical care. The results demonstrate clinical evidence of significant MDIs (quinolones, benzodiazepines, opiate agonists and partial agonists) among MMTP patients in Taiwan. Given the potential adverse effects from co-usage of methadone with benzodiazepines or opiate partial agonists, clinicians should be cautious in prescribing these medications to MMTP patients and incorporate clinical monitoring of potential ADRs of MDIs into treatment plans.

To protect the privacy of MMTP patients, it is difficult to interconnect medical data from different sources. However, the incumbent agency should be responsible for developing pharmacovigilant measures to improve the quality of essential MMTP care services and to prevent the MDIs from occurring. Meanwhile, it is recommended that physicians should check the past and current medication history of MMTP patients, especially who have ever used quinolones, benzodiazepines, opiate agonists and partial agonists. From the findings in this paper, it is desperately needed to provide proper training to prescribing clinicians/providers in MMTPs and generalists in primary care to pay special attention when prescribing methadone and concomitant medications for co-occurring disorders. At the same time, it is also imperative to educate patients and their families of the danger of methadone drug interactions and methadone overdose.

In the future, it will be of interest to look into the association between the patients' doses and genetic polymorphism that affects methadone metabolism, as well as their impacts on the frequency or severity of MDIs.

## Competing interests

The authors declare that they have no competing interests.

## Authors' contributions

HYL helped the corresponding author in designing the study, collecting data, formulating tables and figure as well as interpreting data to write the draft. JHL is the corresponding author who conducted and oversaw this collaborative study. During the study period, he integrated all the data from different sources and communicated with all co-authors to analyze and interpret the results. LTW assisted our team in providing valuable suggestions and revising the contents of this paper. JSW was responsible for collecting the clients' data from the National Health Insurance (NHI) program. CFY and HPT are psychiatrists who helped recruited clients from clinics. All authors read and approved the final manuscript.
